# Past and Future of Non-*Saccharomyces* Yeasts: From Spoilage Microorganisms to Biotechnological Tools for Improving Wine Aroma Complexity

**DOI:** 10.3389/fmicb.2016.00411

**Published:** 2016-03-31

**Authors:** Beatriz Padilla, José V. Gil, Paloma Manzanares

**Affiliations:** ^1^Departament de Bioquímica i Biotecnologia, Facultat d’Enologia, Universitat Rovira i VirgiliTarragona, Spain; ^2^Departamento de Biotecnología de Alimentos, Instituto de Agroquímica y Tecnología de Alimentos, Consejo Superior de Investigaciones CientíficasPaterna, Spain; ^3^Departamento de Medicina Preventiva y Salud Pública, Ciencias de la Alimentación, Toxicología y Medicina Legal, Facultad de Farmacia, Universitat de ValènciaBurjassot, Spain

**Keywords:** non-*Saccharomyces* yeasts, enzymes, secondary metabolites, primary aroma, secondary aroma, mixed starters, aroma complexity

## Abstract

It is well established that non-*Saccharomyces* wine yeasts, considered in the past as undesired or spoilage yeasts, can enhance the analytical composition, and aroma profile of the wine. The contribution of non-*Saccharomyces* yeasts, including the ability to secret enzymes and produce secondary metabolites, glycerol and ethanol, release of mannoproteins or contributions to color stability, is species- and strain-specific, pointing out the key importance of a clever strain selection. The use of mixed starters of selected non-*Saccharomyces* yeasts with strains of *Saccharomyces cerevisiae* represents an alternative to both spontaneous and inoculated wine fermentations, taking advantage of the potential positive role that non-*Saccharomyces* wine yeast species play in the organoleptic characteristics of wine. In this context mixed starters can meet the growing demand for new and improved wine yeast strains adapted to different types and styles of wine. With the aim of presenting old and new evidences on the potential of non-*Saccharomyces* yeasts to address this market trend, we mainly review the studies focused on non-*Saccharomyces* strain selection and design of mixed starters directed to improve primary and secondary aroma of wines. The ability of non-*Saccharomyces* wine yeasts to produce enzymes and metabolites of oenological relevance is also discussed.

## Introduction

Wine fermentation is a complex microbiological process in which yeasts play a fundamental role. Although *Saccharomyces cerevisiae* is the main microorganism involved in the alcoholic fermentation of grape must, winemaking is a non-sterile process. Many other species of yeasts belonging to various non-*Saccharomyces* genera occur in grape juice and contribute to the first stages of fermentation and to the organoleptic characteristics of final wine (Fleet, 2008).

In the past, non-*Saccharomyces* yeasts were considered to be of secondary significance or undesirable spoilage yeasts; nowadays it is widely accepted that selected strains through appropriate screenings can positively impact on the winemaking process. Thus the growing demand for new and improved wine yeast strains adapted to different types and styles of wines can be met by non-*Saccharomyces* wine yeasts. Since these yeasts are in general poor fermenters, the design of mixed starters including selected non-*Saccharomyces* with optimized biotechnological characteristics and *S. cerevisiae* to ensure a complete fermentation has become one of the main challenges of researchers and oenologists. Moreover, proper mixed starter management during fermentation will allow winemakers to tailor wines to the changing demands of consumers.

The production of wines with particular flavor profiles has been one of the main reasons for including non-*Saccharomyces* yeasts in mixed starters. However, promising approaches to lowering alcohol content of wines, to control wine spoilage or to improve oenological properties are being explored, and undoubtedly they represent new opportunities for exploitation in wine production. Here we revisit the contribution of non-*Saccharomyces* yeasts to wine aroma complexity. First we review the ability of these yeasts to produce enzymes and metabolites of oenological relevance and finally we discuss the design of mixed starters directed to improve primary and secondary aroma of wines. Special attention was paid to update the information covered in recent reviews on the impact of non-*Saccharomyces* in wine production.

### Non-*Saccharomyces* Yeasts in Wine Production

In the second half of the 19th century, Louis Pasteur revealed the role of yeasts during the wine fermentation process, demonstrating that yeast is the primary catalyst responsible for the conversion of grape sugars to alcohol and CO_2_. He noticed that in fermenting grape musts coexisted a wide variety of microorganisms, including different types of yeasts. His drawings, based on microscopic observations, showed two kinds of yeasts. The first, which was abundant in the early stages of the process, was the small, apically budding, lemon-shaped *Saccharomyces apiculatus* (now *Hanseniaspora uvarum*). The second which became the most abundant as alcoholic fermentation progressed, was a larger yeast with round cells, which Pasteur called either *Saccharomyces pastorianus* or *Saccharomyces ellipsoideus* (probably the current *S. cerevisiae*) (Barnett, 2000).

Despite the complex wine microbial ecology, *S. cerevisiae* became the wine yeast *par excellence* based mainly on its fermentation behavior (Reed and Peppler, 1973; Bely et al., 1990; Fleet, 1993), but also on its important role in the release of aroma precursors (Dubourdieu, 1996; Úbeda and Briones, 2000; Ugliano et al., 2006) and in the formation of secondary aroma (Fleet, 1993; Pretorius, 2003). The other yeast species occurring in musts and wines were considered as a source of potential spoilage problems during wine production. In fact, the presence or overgrowth of some of these species was often related to stuck or sluggish fermentations, or to the production of detrimental compounds to the sensory properties of wine (du Toit and Pretorius, 2000). In the context of this simplistic view of the wine fermentation process, where the most important objective was the inoculation and dominance of *S. cerevisiae*, the term ‘non-*Saccharomyces’* yeasts referred to the wide variety of yeast genera, including more than 20 in both Ascomycota and Basidiomycota phyla, present in grape juice.

Yeasts occurring in grape musts at the early stages of fermentation originate from two main sources, the vineyard and the grapes, and the contact surfaces and equipment of the winery (Pretorius et al., 1999). The latter plays a small role as a source of non-*Saccharomyces* yeasts, while *S. cerevisiae* is the predominant yeast in such surfaces (Peynaud and Domercq, 1959; Rosini, 1984; Lonvaud-Funel, 1996; Pretorius, 2000). However, it has been recently reported for the first time the implantation in grape must of *Hanseniaspora* species present in the winery environment (Grangeteau et al., 2015) opening the possibility, still unexplored, that some of the non-*Saccharomyces* species could persist from 1 year to another in the winery environment and become dominant during fermentation, as usually described for *S. cerevisiae* (Santamaría et al., 2005; Le Jeune et al., 2006; Mercado et al., 2007).

The great quantitative and qualitative variability of non-*Saccharomyces* species found in the early stages of fermentation can be explained by the large number of factors influencing the grape microbiota such as localization, climatic conditions, cultivar, application of pesticides, and other agronomic practices, stage of ripening, health of the grapes, harvesting procedures and the specific weather conditions in each vintage year (Martini et al., 1980; Rosini et al., 1982; Querol et al., 1990; Regueiro et al., 1993; Epifanio et al., 1999; Jolly et al., 2006; Brilli et al., 2015). In spite of this wide variability of yeast species, during the first 3–4 days of a spontaneous fermentation of grape must, yeast population is numerically dominated by apiculate yeasts, *Hanseniaspora*/*Kloeckera*, and *Candida* species, followed by several species belonging to the genera *Metschnikowia* and *Pichia*, and occasionally to *Brettanomyce*s, *Kluyveromyces, Schizosaccharomyces, Torulaspora, Rhodotorula, Zygosaccharomyces*, and *Cryptococcus* genera (Goto, 1980; Benda, 1982; Fleet et al., 1984; Heard and Fleet, 1985; Parish and Caroll, 1985; Martínez et al., 1989; Herraiz et al., 1990; Frezier and Dubourdieu, 1992; Schütz and Gafner, 1993; Granchi et al., 1998; Combina et al., 2005; Fleet, 2008). This scenario, with abundance of apiculate yeasts in the 1st days of alcoholic fermentation and varying amounts of other non-*Saccharomyces* yeasts, followed by the progressive dominance of *S. cerevisiae* is a common denominator in the process of elaboration of all wines, including those produced by inoculation with selected wine yeast strains (Heard and Fleet, 1985).

Industrial wine fermentations are currently conducted by starters of selected wine yeast strains of *S. cerevisiae*. The first reported use of a selected yeast starter for wine production dates from 1890, when Müller-Thurgau introduced this technology adapting the techniques developed by Christian Hansen for the Carlsberg Brewery (Pretorius, 2000; Barnett and Lichtenthaler, 2001). Nowadays, the use of active dry yeasts is one of the most common practices in winemaking and the market offers a wide variety of yeast strains as dehydrated cultures promising a good implantation, specific skills for different types of wines and a great list of other features such as the ability of enhancing varietal and fermentative aromas, glycerol production, tolerance to alcohol, or specific enzymatic activities. However, the main reason of selected starters is to achieve wines with uniform quality through different years avoiding the variability associated with spontaneous fermentations and the risk of spoilage (Beltran et al., 2002; Santamaría et al., 2005). In such cases, dominant growth of the inoculated strain is required. However many factors might affect the implantation/persistence of individual strains within the total population (Fleet, 2008; Blanco et al., 2012), including the variability that exists from one vintage to another at a given winery (Lange et al., 2014).

**FIGURE 1:**
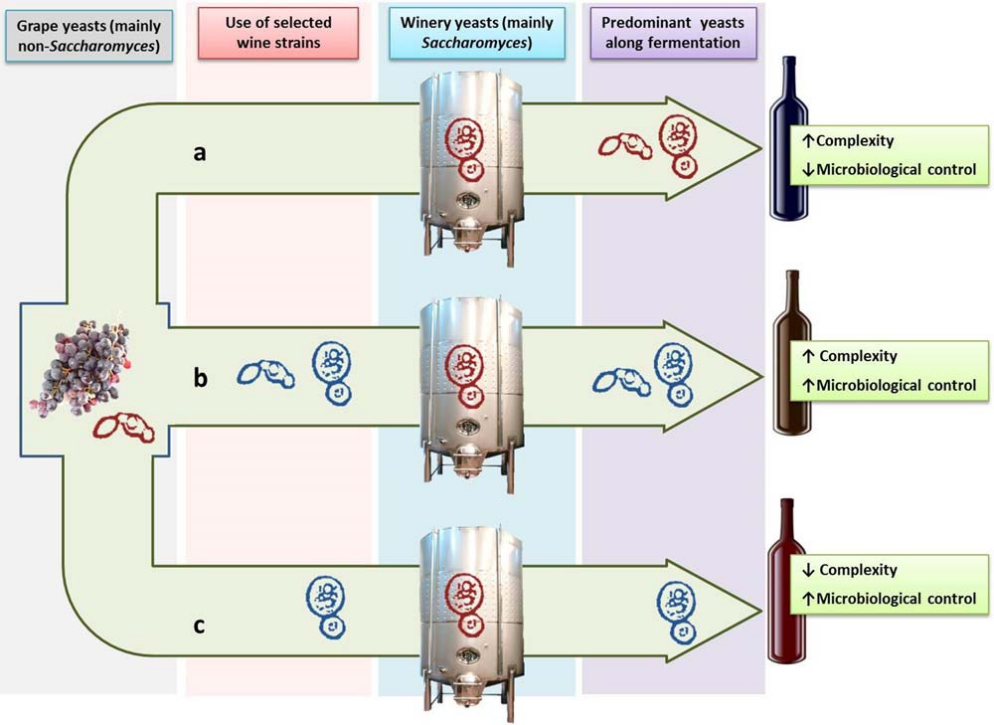
**Use of selected strains of *Saccharomyces* and non-*Saccharomyces* yeasts in winemaking.** Spontaneous fermentation **(a)** allows the development of indigenous yeasts from grapes (mainly non-*Saccharomyces*) and winery (mainly *Saccharomyces*) leading to wines with a greater aromatic complexity but with less microbiological control. Inoculation with a selected strain of *Saccharomyces cerevisiae* **(c)** leads to a greater microbiological control but can reduce the aromatic complexity of wine. The use of mixed cultures of *S. cerevisiae* and non-*Saccharomyces* selected strains **(b)** allows to obtain wines with both greater aromatic complexity and microbiological control of the process. Autochthonous and inoculated selected yeasts are represented in red and blue color, respectively.

Despite the advantages of using pure cultures of *S. cerevisiae* with regard to the easy of control and homogeneity of fermentations, wine produced with pure yeast monocultures lacks the complexity of flavor, stylistic distinction and vintage variability caused by indigenous yeasts (Lambrechts and Pretorius, 2000; Romano et al., 2003). This fact is a never-ending debate between researchers and oenologists, and the growth of non-*Saccharomyces* yeasts can still be seen as an uncontrollable risk or as an opportunity of improving the quality of wine. Nevertheless it is worthwhile to note that the world’s best quality wines are produced after a fermentation process in which, in a greater or lesser extent, various species of non-*Saccharomyces* yeasts have played a role in the winemaking process and, therefore, have contributed to the final result. It is in this context where the inclusion of non-*Saccharomyces* wine yeast species as part of mixed starters together with *S. cerevisiae* to improve wine quality was suggested as a way of taking advantage of spontaneous fermentations without running the risks of stuck fermentations or wine spoilage (Jolly et al., 2003; Rojas et al., 2003; Romano et al., 2003; Ciani et al., 2006). However this practice is linked to new challenges for researchers and oenologists such as the selection of suitable non-*Saccharomyces* strains, the appropriate modality and time of inoculation, the proportion of yeasts in the culture and the potential microorganism interactions, among others. **Figure 1** shows a schematic outline of spontaneous versus inoculated fermentation and the use of mixed starters of selected non-*Saccharomyces* yeasts with strains of *S. cerevisiae* as an alternative to both approaches.

### Influence of Non-*Saccharomyces* Yeasts on Wine Aroma

Undoubtedly, aroma is one of most important characteristics that contribute to the quality of wine. As in many foods, wine aroma is composed by 100s of different compounds with concentrations that can vary between 10^-1^ and 10^-10^ g/kg (Rapp and Mandery, 1986). The balance and interaction of all of them determine the wine aromatic quality.

Wine aroma can be subdivided into three groups: the varietal or primary aroma, determined by the grape variety; the fermentation or secondary aroma; and the bouquet or tertiary aroma resulting from the transformation of aromas during aging. Non-*Saccharomyces* yeasts can influence both the primary and secondary aroma through the production of enzymes and metabolites, respectively.

### Influence on Primary Aroma

Primary or varietal aroma is formed during the ripening of grapes and its contribution to the final wine aroma is considered an appreciated feature. The production of active compounds of primary wine odor takes place in the exocarp of the grape berry and its final concentration in wine is primarily influenced by the vine variety and secondarily by the state of ripeness and the agronomic and oenological practices (Ewart et al., 1985; Spayd et al., 2002; Hernández-Orte et al., 2008, 2015).

Compounds forming primary aroma belong to a limited number of chemical families, including methoxypyrazines, C_13_-norisoprenoids, volatile sulfur compounds, and terpenes (Ebeler and Thorngate, 2009). Methoxypyrazines are products of amino acid metabolism, and they have been associated to vegetal, green, and herbaceous aromas in certain vine cultivars (reviewed in Sidhu et al., 2015). C_13_-norisoprenoids derive from carotenoids and particularly β-ionone and β-damascenone are considered impact volatiles of non-floral grapes (Fang and Qian, 2006; Bindon et al., 2007; Pineau et al., 2007; Ristic et al., 2010; Fang and Qian, 2016). Certain organic volatile sulfur compounds such as aromatic thiols make important contributions to Sauvignon Blanc and red cultivars aroma (Darriet et al., 1995; Tominaga et al., 1996, 1998a; Bouchilloux et al., 1998), whereas terpenoids, although present in grapes of all vine varieties, occur in aromatic varieties such as Muscat, Gewürztraminer and Rhine Riesling in the highest concentrations (King and Dickinson, 2000). In grape berries and corresponding wines, approximately seventy terpenoid compounds have been identified (Mateo and Jiménez, 2000). Among them, five monoterpenoid alcohols, namely linalool, geraniol, nerol, citronellol, and α-terpineol are the most abundant and the strongest contributors to wine aroma (Rapp, 1998; Mateo and Jiménez, 2000; Carrau et al., 2005). These compounds provide floral notes and have low odor thresholds (Zalacain et al., 2007).

**FIGURE 2:**
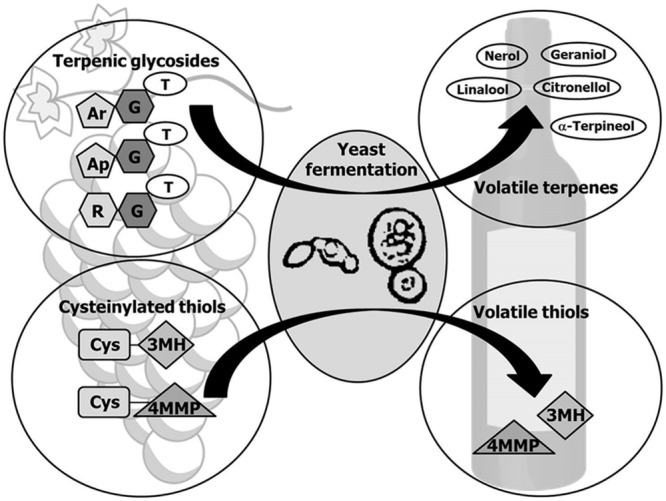
**Release of primary aroma compounds by yeasts.** Monoterpenes and volatile thiols occur in grape as odorless precursors which can be released by enzymatic activities of *Saccharomyces* and non-*Saccharomyces* yeasts during fermentation. Monoterpene glycosides are mainly glucosides and diglycosides, in which the glucose moiety (G) has been further substituted mainly with arabinose (Ar), apiose (Ap), or rhamnose (R). A two-step enzyme-catalyzed reaction is the main mechanism proposed for the enzymatic hydrolysis of the diglycosides and subsequent release of the free volatile terpene (T) to wine. First a specific glycosidase cleaves the linkage between both sugars and in a second step the released glucoside is hydrolyzed by a β-D-glucosidase, liberating glucose and the corresponding terpene. Volatile thiols are generated from the odourless cysteinylated precursors cysteine-3-mercaptohexan-1-ol (Cys-3MH) and cysteine-4-mercapto-4-methylpentan-2-one (Cys-4MMP) by the action of carbon-sulfur-lyases.

Interestingly most of primary aroma compounds are found in free or bound forms. The latter are not odorant compounds which hydrolysis can occur during fermentation through the action of wine yeasts (**Figure 2**). Particularly important are aroma precursors linked to sugar molecules, mainly terpenol and C_13_-norisoprenoid glycosides, and the non-volatile precursor forms of volatile thiols conjugated to cysteine or glutathione. The main yeast enzymes involved in the release of aroma compounds from odorless grape precursors are glycosidases that hydrolyze the non-volatile glycosidic precursors (Gunata et al., 1988), and carbon-sulfur lyases that release volatile thiols from aroma-inactive cysteine-bound conjugates (Tominaga et al., 1998b). Below we focus on the production of these enzymes of oenological relevance by non-*Saccharomyces* wine yeasts. **Table 1** summarizes the yeast species described as producers of glycosidases and carbon-sulfur lyases.

**Table 1:** Non-*Saccharomyces* yeast species described as producers of enzymes involved in the release of aroma compounds from grape precursors.

	Enzyme^1^				
Yeast species	BGL	ARA	RHA	XYL	CSL	Reference
*A. pullulans*	x	x	x			McMahon et al., 1999
*B. anomalus*	x					Fia et al., 2005
*Brettanomyces* spp.	x					Cordero-Otero et al., 2003; Arévalo-Villena et al., 2007
*C. guilliermondii*	x		x	x		McMahon et al., 1999; Cordero-Otero et al., 2003; Rodríguez et al., 2004
*C. molischiana*	x					Fernández-González et al., 2003; Genovés et al., 2003
*C. stellata*	x		x	x		Rosi et al., 1994; Strauss et al., 2001; Cordero-Otero et al., 2003
*C. utilis*				x		Yanai and Sato, 2001
*C. zemplinina*					x	Anfang et al., 2009
*D. castellii*	x					Rosi et al., 1994
*D. hansenii*	x					Rosi et al., 1994; Yanai and Sato, 1999; Fernández-González et al., 2003
*D. polymorphus*	x					Rosi et al., 1994; Cordero-Otero et al., 2003; Arévalo-Villena et al., 2007
*D. pseudopolymorphus*	x					Cordero-Otero et al., 2003; Arévalo-Villena et al., 2006, 2007
*D. vanriji*	x					García et al., 2002
*Hanseniaspora* sp.	x			x		Swangkeaw et al., 2011
*H. guilliermondii*	x					Manzanares et al., 2000
*H. osmophila*	x			x		Manzanares et al., 1999, 2000
*H. vineae*	x	x	x	x		Mateo et al., 2011; Maturano et al., 2012; López et al., 2015
*H. uvarum*	x	x	x	x		Rosi et al., 1994; Charoenchai et al., 1997; Manzanares et al., 1999, 2000; Strauss et al., 2001; Fernández-González et al., 2003; Rodríguez et al., 2004; Arévalo-Villena et al., 2007; Mateo et al., 2011; López et al., 2015
*I. terricola*	x					González-Pombo et al., 2011
*K. thermotolerans*	x				x	Rosi et al., 1994; Zott et al., 2011
*M. pulcherrima/C. pulcherrima*	x			x	x	Rosi et al., 1994; Fernández-González et al., 2003; Rodríguez et al., 2004, 2010a; González-Pombo et al., 2008; Zott et al., 2011
*P. angusta*			x			Yanai and Sato, 2000a
*P. anomala*	x	x	x	x		Rosi et al., 1994; Charoenchai et al., 1997; Manzanares et al., 1999, 2000; Spagna et al., 2002; Mateo et al., 2011; Swangkeaw et al., 2011
*P. capsulata*		x				Yanai and Sato, 2000b
*P. guilliermondii*			x			Rodríguez et al., 2004, 2010b
*P. kluyvery*					x	Anfang et al., 2009
*P. membranifaciens*	x			x		López et al., 2015
*S. ludwigii*	x					Rosi et al., 1994
*S. pombe*	x					Rosi et al., 1994
*S. pararoseus*	x					Baffi et al., 2013
*T. delbrueckii*	x				x	Hernández-Orte et al., 2008; Zott et al., 2011; Maturano et al., 2012; Cordero-Bueso et al., 2013; Ĉuŝ and Jenko, 2013
*T. asahii*	x					Wang et al., 2011
*W. anomalus*	x	x		x		Sabel et al., 2014; López et al., 2015
*Z. bailii*	x					Rosi et al., 1994; Cordero-Otero et al., 2003

*^1^BGL, β-D-glucosidase; ARA, α-L-arabinofuranosidase; RHA, α-L-rhamnosidase; XYL, β-D-xylosidase; CSL, carbon-sulfur lyase.*

#### Glycosidases

Since the demonstration that the aromatic components of certain grape varieties are present in the grape berry both in free form and bound to sugars as glycosides (Cordonnier and Bayonove, 1974; Williams et al., 1982), there has been a continuous research to find glycosidases able to release varietal aromas from precursors. The bound aroma fraction comprises glucosides and diglycosides, and compounds such as terpenols, terpene diols, 2-phenylethyl alcohol, benzyl alcohol and C_13_-norisoprenoids have been shown to be aglycons of such glycosides (Winterhalter and Skouroumounis, 1997). Diglycosides mainly include 6-O-α-L-arabinofuranosyl-β-D-glucopyranosides, 6-O-α-L-rhamnopyran-osyl-β-D-glucopyranosides and 6-O-β-D-apiofuranosyl-β-D-glucopyranosides. Due to the important role of monoterpenes in determining the aroma of grapes and wines, hydrolysis of terpene glycosides has been the main focus of research. It is now well established that the enzymatic hydrolysis occurs in two steps (Gunata et al., 1988). During the first step and depending on the conjugate, the glycosidic linkage is cleaved by either an α-L-arabinofuranosidase, an α-L-rhamnosidase or a β-D-apiosidase, and the corresponding monoterpenyl-β-D-glucosides are released. In the second step, monoterpenes are liberated by the action of a β-D-glucosidase.

Although oenological yeasts may produce glycosidases, the potential effectiveness of enzymes may be hampered by acidic wine conditions or high ethanol concentrations; another limitation of these enzymes is their weak activity in the presence of glucose in the must or wine, making it especially necessary to analyze their inhibition by these wine components. The potential effectiveness of yeast-derived glycosidases is even further reduced in most cases by the fact that some of the enzymes are intracellular and released only in very small amounts into the culture medium. The degree to which these factors inhibit glycosidase production and activity depends on the species and strains of the organisms involved, pointing out the need of enzyme screenings.

Widespread occurrence of β-D-glucosidase activity in non-*Saccharomyces* yeasts has been revealed in several screenings. Rosi et al. (1994) showed that yeasts of the genera *Candida, Debaryomyces, Hanseniaspora*/*Kloeckera, Kluyveromyces, Metschnikowia, Pichia, Saccharomycodes, Schizosaccharomyces*, and *Zygosaccharomyces* can produce β-D-glucosidases. Later on this capability was confirmed by other authors (Charoenchai et al., 1997; McMahon et al., 1999; Manzanares et al., 2000; Strauss et al., 2001; Spagna et al., 2002; Cordero-Otero et al., 2003; Fernández-González et al., 2003; Rodríguez et al., 2004; González-Pombo et al., 2008; Sabel et al., 2014; López et al., 2015) and was extended also to the genera *Torulaspora* (Hernández-Orte et al., 2008; Cordero-Bueso et al., 2013), *Brettanomyces* (Cordero-Otero et al., 2003; Fia et al., 2005; Arévalo-Villena et al., 2007), and *Trichosporon* (Wang et al., 2011). Some of these enzymes, selected as a result of their activity with artificial substrates, proved also effective in hydrolyzing either a grape glycoside extract or in releasing terpenols after addition to must or wine. *Debaryomyces hansenii* and *H. uvarum* β-D-glucosidases hydrolyzed terpenic glycosides isolated from grape must (Rosi et al., 1994; Fernández-González et al., 2003). Terpene release was also observed in must and wine treated with β-D-glucosidases from *Hanseniaspora* sp. and *Pichia anomala* (Swangkeaw et al., 2011). Moreover, results suggested that the enzyme from *Hansenispora* sp. was more efficient in releasing desirable aromas during an early stage of alcoholic fermentation while β-D-glucosidase from *P. anomala* was suitable at the final stage. Several yeast β-D-glucosidases have been purified and characterized. Two *Debaryomyces* β-D-glucosidases suitable for enhancing wine aroma have been reported. An intracellular *D. hansenii* β-D-glucosidase, tolerant to ethanol and glucose, efficiently released monoterpenols from the glycosides extracted from Muscat grape must. In addition, when the enzyme was added during Muscat fermentation, a considerably increase in the concentration of mainly nerol and linalool was observed (Yanai and Sato, 1999). By contrast, a *Debaryomyces pseudopolymorphus* strain (Cordero-Otero et al., 2003) produced an exocellular β-D-glucosidase with acidic optimal pH and not inhibited by glucose or ethanol (Arévalo-Villena et al., 2006), although the effectiveness of the purified enzyme in winemaking was not tested. Enzymatic treatment of wine with a purified ethanol tolerant β-D-glucosidase from *Sporidiobolus pararoseus*, a member of oenological ecosystems in the southeastern region of Brazil, considerably increased the amount of free terpenes (Baffi et al., 2011, 2013).

Several strategies for either improving β-D-glucosidase stability or enzyme yield have been described. An extracellular β-D-glucosidase from *Issatchenkia terricola*, active in the presence of glucose, ethanol, and metabisulfite was immobilized for improving acidic pH stability. This strategy increased the amount of monoterpenes and norisoprenoids, showing the potential of the immobilized enzyme for aroma development in wines (González-Pombo et al., 2011). With respect to yield enhancement, the usefulness of response surface methodology for optimizing the production of a *Trichosporon asahii* β-D-glucosidase was reported (Wang et al., 2012). *T. asahii* β-D-glucosidase exhibited better ability than fungal and plant commercial enzymes in hydrolyzing aromatic precursors in young wine. Also, a recombinant *S. cerevisiae* wine yeast expressing the *Candida molischiana bgln* gene encoding a β-D-glucosidase able to release terpenols and alcohols from a glycoside extract has been used to facilitate protein purification (Genovés et al., 2003).

Some of the β-D-glucosidase screenings described above also included the search of less common glycosidases, such as α-L-arabinofuranosidase, α-L-rhamnosidase and β-D-xylosidase. From more than 300 wine yeast strains, only one strain of *P. anomala* showed α-L-arabinofuranosidase activity whereas none of them was positive for α-L-rhamnosidase production (Spagna et al., 2002). Also the potential of certain wine yeasts from the genera *Candida, Hanseniaspora*, and *Pichia* to produce β-D-xylosidase activity active at winemaking conditions has been discussed (Manzanares et al., 1999; Yanai and Sato, 2001; Rodríguez et al., 2004; López et al., 2015). Interestingly yeast strains able to display several glycosidase activities have been reported: one strain of *Aureobasidium pullulans* able to hydrolyze grape glycosides displayed β-D-glucosidase, α-L-arabinofuranosidase and α-L-rhamnosidase activities, whereas *Candida guilliermondii* produced both β-D-glucosidase and α-L-rhamnosidase (McMahon et al., 1999). Two *H. uvarum*, one *Hanseniaspora vineae* and one *P. anomala* strains were described as producers of the four glycosidase activities (Mateo et al., 2011), while a *Wickerhamomyces anomalus* (alternative names *Hansenula anomala, P. anomala* and *Candida pelliculosa*) strain producing β-D-glucosidase, also exhibited α-L-arabinofuranosidase and β-D-xylosidase activities (Sabel et al., 2014). However, the effectiveness of purified glycosidases for terpene releasing from precursors has only been reported for the intracellular α-L-rhamnosidases from *Pichia angusta* (Yanai and Sato, 2000a) and *Pichia guilliermondii* (Rodríguez et al., 2004, 2010b), an α-L-arabinofuranosidase from *Pichia capsulata* (Yanai and Sato, 2000b) and a β-D-xylosidase from *Candida utilis* (Yanai and Sato, 2001). The latter also increased the concentration of terpenes after addition to Moscatel grape must during fermentation (Yanai and Sato, 2001).

The role of exo-glucanases in the release of aromatic compounds from glycosidically bound precursors in a single enzymatic step has been discussed (Gil et al., 2005). In this regard, a yeast isolate AS1, identified as a *W. anomalus* strain was selected by its capability to hydrolyze several synthetic and natural glycosides under oenological conditions (Sabel et al., 2014). Later, the enzyme responsible for the hydrolysis of selected glycosides was purified from the culture supernatant of AS1 and characterized as a multifunctional exo-β-1,3-glucanase active under typical wine related conditions (Schwentke et al., 2014).

The feasibility of using β-D-glucosidase-producing yeasts in fermentation instead of adding purified enzymes represents an interesting option. Either alone or in a mixed starter with *S. cerevisiae*, the ability of non-*Saccharomyces* yeasts to contribute to the aromatic wine profile has been assessed. Different strains of *Torulaspora delbrueckii* contributed to the aroma profile with flowery and fruity aroma (Maturano et al., 2012; Cordero-Bueso et al., 2013). Secreted *H. vineae* and *T. delbrueckii* β-D-glucosidases were detected throughout the fermentation process, although activity diminished with increasing fermentation time, suggesting the adverse effect of ethanol (Maturano et al., 2012). Also, isolates of *Pichia membranifaciens, H. vineae, H. uvarum*, and *W. anomalus* showing β-D-glucosidase activity provoked a moderated overall terpene increase when inoculated to final wines (López et al., 2015). The first mixed starters based on non-*Saccharomyces* yeasts able to produce β-D-glucosidase activity were described for *Debaryomyces vanriji* and *D. pseudopolymorphus* (García et al., 2002; Cordero-Otero et al., 2003), and later on for *Candida pulcherrima* (alternative name *Metschnikowia pulcherrima*) (Rodríguez et al., 2010a) and *T. delbrueckii* (Ĉuŝ and Jenko, 2013). Detailed information of these mixed starters will be found in next sections.

#### Carbon-Sulfur Lyases

Some sulfur containing compounds, the so-called volatile or varietal thiols, can contribute to positive fragances such as tropical, passion fruit and guava-like nuances. These compounds considered to be impact odorants in Sauvignon Blanc wines are 4-mercapto-4-methylpentan-2-one (4MMP), reminiscent of box tree, passion fruit, broom, and black current bud; and 3-mercaptohexan-1-ol (3MH) and 3-mercaptohexyl acetate (3MHA), responsible for the passion fruit, grapefruit, and citrus aroma. Volatile thiols are not unique to Sauvignon Blanc wines. They have been also found to contribute significantly to the aroma profiles of wines made from other varieties such as Riesling, Colombard, Semillon, Cabernet Sauvignon, and Merlot (revised in Coetzee and du Toit, 2012).

Volatile thiols are mostly non-existent in grape juice and they are generated during the fermentation process by yeasts from odorless, non-volatile precursors initially present in must (revised in Cordente et al., 2012). It has been shown that 4MMP and 3MH exist in grapes in their non-volatile precursor form, conjugated to cysteine, or glutathione. The key enzyme for cleaving cysteinylated precursors is the *S. cerevisiae* β-lyase IRC7, with a substrate preference for cys-4MMP over cys-3MH (Roncoroni et al., 2011). The mechanism by which the glutathionated thiol precursors are degraded has not been fully elucidated, but is likely to involve a multi-step pathway with the production of the cysteinylated form as an intermediate (Grant-Preece et al., 2010). No precursor of 3MHA has been identified in grapes; this compound is formed during fermentation through esterification of 3MH by the alcohol acetyltransferase ATF1 (Swiegers et al., 2006).

Undoubtedly the main factor in volatile thiol release during alcoholic fermentation is the yeast strain (Dubourdieu et al., 2006). It was found that *S. cerevisiae* strains varied significantly in terms of their capabilities to produce volatile thiols and to modulate the varietal characters of Sauvignon Blanc wine (Swiegers et al., 2009). With regard to non-*Saccharomyces* species, only two screenings have addressed their feasibility to release volatile thiols. The first screening by Anfang et al. (2009) showed that most of the eleven non-*Saccharomyces* isolates tested were able to produce concentrations of 3MH above the perception threshold, but only two isolates of *Pichia kluyvery* and *Candida zemplinina* (alternative names *Candida stellata* and *Starmerella bacillaris*) produced concentrations of 3MH and 3MHA comparable with those produced by *S. cerevisiae*. In contrast to that found for *S. cerevisiae*, results showed an inverse correlation between the concentrations of 3MH and 3MHA produced by the *P. kluyvery* and *C. zemplinina* isolates, suggesting a decreased ability to convert 3MH to 3MHA, or possibly alternate metabolic routes for its formation (Anfang et al., 2009). In a second screening, the potential impact of 15 non-*Saccharomyces* strains from seven species on 4MMP and 3MH release in model medium and Sauvignon Blanc must was evaluated after partial fermentation (Zott et al., 2011). In general, non-*Saccharomyces* strains had greater ability to release 3MH than 4MMP in both media. Only *M. pulcherrima* and *H. uvarum* strains in model medium and *Kluyveromyces thermotolerans* in must were able to produce significant amounts of 4MMP. With respect to 3MH release, *M. pulcherrima* and *T. delbrueckii* strains released large amounts of this compound in model medium whereas *M. pulcherrima* and *K. thermotolerans* stood out as good producers in natural must. *C. zemplinina* isolates included in the screening did not produce volatile thiols, in contrast to previous results (Anfang et al., 2009). This can be explained by the strain dependent capacity to release 3MH as showed for *M. pulcherrima* (Zott et al., 2011). Undoubtedly additional screening experiments including numerous non-*Saccharomyces* strains are required to obtain a clear image of species-associated behavior or strain effects. Mixed fermentations with volatile thiol releasing yeasts will be discussed in later sections.

### Influence on Secondary Aroma

Most of the compounds that determine wine aroma arise from the fermentation process. Their concentrations are mainly dependent on the predominant yeasts and the fermentation conditions (Egli et al., 1998; Henick-Kling et al., 1998; Steger and Lambrechts, 2000). Although ethanol, glycerol, and CO_2_ are quantitatively the most abundant of these compounds, their contribution to the secondary aroma is relatively limited. Volatile fatty acids, higher alcohols, esters, and, to a lesser extent, aldehydes, have a greater contribution to secondary aroma (Rapp and Versini, 1991), although volatiles derived from fatty acids and from nitrogen- or sulfur-containing compounds also contribute (Boulton et al., 1996). The biosynthesis of these compounds has been reviewed in greater detail by Lambrechts and Pretorius (2000). It is worthwhile to note that the biosynthesis of these compounds is species- and strain-dependent, allowing the selection of those strains of biotechnological interest. Moreover, and depending on the concentration reached in wine, those compounds arising from yeast metabolism have a positive or negative impact on wine aroma and quality. Below we describe the contribution of non-*Saccharomyces* yeast species to wine secondary aroma. **Table 2** shows the yeast species described as high- or low-producers of secondary aroma compounds.

**Table 2:** Secondary aroma compounds produced by non-*Saccharomyces* wine yeasts.

Compound	High producers	Low producers	Reference
**Volatile fatty acids**
Acetic acid	*Hanseniaspora* *Zygosaccharomyces* *S. pombe*	*T. delbrueckii* *K. thermotolerans* *C. stellata/C. zemplinina*	Gallander, 1977; Snow and Gallander, 1979; Ciani and Maccarelli, 1998; du Toit and Pretorius, 2000; Soden et al., 2000; Loureiro and Malfeito-Ferreira, 2003; Romano et al., 2003; Kapsopoulou et al., 2005; Mendoza et al., 2007; Renault et al., 2009; Comitini et al., 2011; Rantsiou et al., 2012; Benito et al., 2014a; Englezos et al., 2015
**Higher alcohols**	*M. pulcherrima* *C. zemplinina* *L. thermotolerans*	*Hanseniaspora* *Zygosaccharomyces*	Romano and Suzzi, 1993; Rojas et al., 2003; Clemente-Jiménez et al., 2004; Moreira et al., 2008; Viana et al., 2008; Andorrà et al., 2010; Beckner Whitener et al., 2015
**Esters**	*Candida* *Hansenula* *Pichia* *Hanseniaspora* *Rhodotorula* *T. delbrueckii* *K. gamospora*		Ough et al., 1968; Suomalainen and Lehtonen, 1979; Nykänen, 1986; Mateo et al., 1991; Sponholz, 1993; Romano et al., 1997; Rojas et al., 2001, 2003; Moreira et al., 2005; Viana et al., 2008; Beckner Whitener et al., 2015
**Aldehydes**
Acetaldehyde		*K. apiculata* *C. krusei* *C. stellata* *H. anomala* *M. pulcherrima* *H. uvarum*	Fleet and Heard, 1993; Romano et al., 2003
**Volatile phenols**	*Brettanomyces/Dekkera* *P. guilliermondii*	*Candida* *K. lactis* *T. delbrueckii* *M. pulcherrima* *H. guilliermondii* *H. osmophila* *P. membranifaciens*	Lambrechts and Pretorius, 2000; Shinohara et al., 2000; Dias et al., 2003; Viana et al., 2008; Renault et al., 2009; Beckner Whitener et al., 2015
**Sulfur compounds**	*Candida* *Hanseniaspora* *T. delbrueckii* *K. gamospora*		Strauss et al., 2001; Moreira et al., 2008; Viana et al., 2008; Renault et al., 2009; Beckner Whitener et al., 2015

#### Volatile Fatty Acids

Acetic acid is responsible for 90% of the volatile acidity of wines while the remaining fatty acids, such as propanoic and butanoic acid, are present in small quantities (Radler, 1993). Their production is also associated with bacterial growth (Ribereau-Gayon et al., 1998). Acetic acid becomes unpleasant at concentrations near its flavor threshold of 0.7–1.1 g/L and usually values between 0.2 and 0.7 g/L are considered optimal (Lambrechts and Pretorius, 2000).

Studies of acetic acid production by non-*Saccharomyces* yeasts have generated highly variable results. Some non-*Saccharomyces* genera such as *Hanseniaspora* and *Zygosaccharomyces* have been traditionally described as producers of excessive amounts of acetic acid (du Toit and Pretorius, 2000; Loureiro and Malfeito-Ferreira, 2003; Romano et al., 2003; Mendoza et al., 2007) and, for this reason, they have been considered for long time as spoilage yeasts. Also the species *Schizosaccharomyces pombe* is commonly associated with high levels of acetic acid (Gallander, 1977; Snow and Gallander, 1979). However, this compound is produced with a considerably strain variability. For instance, levels of acetic acid ranging from about 0.6 g/L to more than 3.4 g/L have been described for *H. uvarum* strains (Romano et al., 2003) while a screening of *S. pombe* allowed the selection of strains producing less than 0.4 g/L of acetic acid (Benito et al., 2014a).

By contrast, different screenings of *T. delbrueckii* strains for desirable oenological properties pointed out differences in fermentative capability but always a low production of volatile acidity when compared to *S. cerevisiae* (Ciani and Maccarelli, 1998; Renault et al., 2009; Comitini et al., 2011). This feature is also a characteristic of *Lachancea thermotolerans* (previously known as *K. thermotolerans*) together with the high production of L-lactic acid (Kapsopoulou et al., 2005). *C. stellata/C. zemplinina* presents a strong fructophilic character (Soden et al., 2000), which may be an advantage during the fermentation of sweet wines, since this species do not produce excessive levels of acetic acid as a response to the osmotic stress in comparison to *S. cerevisiae* (Rantsiou et al., 2012). Recently the strong fructophilic character of *C. zemplinina* and its ability to produce low quantities of ethanol and acetic acid and high amounts of glycerol were confirmed (Englezos et al., 2015).

#### Higher Alcohols

They are the largest group of aromatic compounds (Amerine et al., 1980). Higher alcohols contribute to the aromatic complexity of wine at concentrations below 300 mg/L. However when their concentrations exceed 400 mg/L, they are considered to have a negative effect on aroma (Rapp and Mandery, 1986). The importance of higher alcohols is also related to their role as ester precursors (Soles et al., 1982).

In general, studies of higher alcohol production in non-*Saccharomyces* yeasts highlight the influence that these yeasts can have on the chemical composition and quality of wine (Herraiz et al., 1990; Mateo et al., 1991; Gil et al., 1996). In fermented musts, the total production of higher alcohols by pure cultures of *Hanseniaspora* species is lower than that found with *S. cerevisiae* (Rojas et al., 2003; Moreira et al., 2008; Viana et al., 2008). Also, *Zygosaccharomyces* strains isolated from grape musts have been described as producers of low amounts of higher alcohols (Romano and Suzzi, 1993). By contrast, *C. zemplinina* wines contained huge amounts of higher alcohols, which concentrations clearly exceeded 400 mg/L (Andorrà et al., 2010).

Regarding specific alcohols, increased production of 2-phenylethyl alcohol, compound associated with pleasant aromas, has been described as a characteristic of *M. pulcherrima* (Clemente-Jiménez et al., 2004), *L. thermotolerans* (Beckner Whitener et al., 2015), and *C. zemplinina* (Andorrà et al., 2010).

#### Esters

Esters are the most abundant compounds found in wine, with around 160 identified to date. Although various esters can be formed during fermentation, the most abundant are those derived from acetic acid (ethyl acetate, isoamyl acetate, isobutyl acetate, and 2-phenylethyl acetate) and ethyl esters of saturated fatty acids (ethyl butanoate, ethyl caproate, ethyl caprylate, and ethyl caprate). The main ester in wine is ethyl acetate, and it can impart spoilage character at levels of 150–200 mg/L (Lambrechts and Pretorius, 2000).

Non-*Saccharomyces* wine yeasts, known as good producers of esters, have been traditionally associated with the negative effects of high ethyl acetate formation, whereas the levels of ethyl esters produced by these yeasts are generally much lower than those detected in *S. cerevisiae* wines (Rojas et al., 2001, 2003).

Species belonging to the genera *Candida, Hansenula*, and *Pichia* were described as having a greater capacity to produce ethyl acetate than wine strains of *S. cerevisiae* (Ough et al., 1968; Nykänen, 1986). Also, in a study where ester production was grouped by yeast genera, *Hanseniaspora* and *Pichia* stood out by the production of ethyl acetate (Viana et al., 2008). Both genera produced similar ethyl acetate levels, but *Hanseniaspora* was also a potent producer of specific fruity acetate esters such as 2-phenylethyl acetate and isoamyl acetate (Rojas et al., 2001; Moreira et al., 2005; Viana et al., 2008), whereas the genera *Pichia* and *Rhodotorula* produced remarkable levels of isoamyl acetate (Suomalainen and Lehtonen, 1979; Viana et al., 2008). Among *Hanseniaspora* species, specifically *H. uvarum* is reported to be a good producer of esters in general (Mateo et al., 1991; Sponholz, 1993; Romano et al., 1997) whereas *Hanseniaspora guilliermondii* and *Hanseniaspora osmophila* are strong producers of 2-phenylethyl acetate (Rojas et al., 2001, 2003; Viana et al., 2008).

Regarding ethyl esters, production of ethyl caprylate seems to be a characteristic of *T. delbrueckii* (Viana et al., 2008). The aroma profile of the newly discovered yeast *Kazachstania gamospora* showed that this species produced more esters than the *S. cerevisiae* control strain, but specially phenylethyl propionate, an ester desirable in wine due to its floral aroma (Beckner Whitener et al., 2015).

#### Aldehydes

These compounds with apple-like odors are important to the aroma and bouquet of wine due to their low sensory threshold values. Among aldehydes, acetaldehyde constitutes more than 90% of the total content of wines, and its amount can vary from 10 mg/L up to 300 mg/L (Lambrechts and Pretorius, 2000).

*Saccharomyces cerevisiae* strains usually produce higher acetaldehyde levels (5–120 mg/L) than non-*Saccharomyces* species (up to 40 mg/L) such as *Kloeckera apiculata, Candida krusei, C. stellata, H. anomala*, and *M. pulcherrima* (Fleet and Heard, 1993). A mean acetaldehyde concentration of around 25 mg/L was described for *H. uvarum* strains, although significant differences in production among strains were observed (Romano et al., 2003).

#### Volatile Phenols and Sulfur Compounds

Among volatile phenols, the most important are vinylphenols in white wines and ethylphenols in red wines. Their presence is always undesirable, since even at concentrations below the perception threshold they are reported to mask the fruity notes of white wines. These compounds are produced from the non-volatile ferulic and *p*-coumaric acids. Traditionally, ethylphenol producers have been ascribed to the genus *Brettanomyces*/*Dekkera* (Lambrechts and Pretorius, 2000). However, several studies also identified *Candida* species, *Kluyveromyces lactis, T. delbrueckii, M. pulcherrima*, and *P. guilliermondii* strains as volatile phenol producers, although only *P. guilliermondii* displayed the same conversion capacity as *Dekkera* species (Shinohara et al., 2000; Dias et al., 2003; Renault et al., 2009; Beckner Whitener et al., 2015). By contrast *H. guilliermondii, H. osmophila*, and *P. membranifaciens* were not able to decarboxylate either ferulic or *p*-coumaric acids (Viana et al., 2008).

The sensory properties of sulfur compounds vary extensively and although most of them are associated with negative aromatic descriptors, they can have a positive contribution to wine aroma through the introduction of fruity notes (reviewed in Swiegers and Pretorius, 2005). The main compound in this group is hydrogen sulfide. Production by non-*Saccharomyces* yeasts includes *Candida* and *Hanseniaspora* species (Strauss et al., 2001; Viana et al., 2008) as well as *T. delbrueckii* (Renault et al., 2009). The contribution of *H. uvarum* and *H. guilliermondii* to the sulfur compound profile of wines was evaluated by Moreira et al. (2008) and it was concluded that the growth of the apiculate yeasts might not have a negative influence. It should also be noted that *T. delbrueckii* and *K. gamospora* are able to produce the sulfur compound 3-methylthio-1-propanol in higher concentration than *S. cerevisiae*, although it was dependent on the must variety (Beckner Whitener et al., 2015). The formation of volatile thiols by non-*Saccharomyces* yeasts has been described in the primary aroma section.

## Mixed Starters

The use of mixed starters of selected non-*Saccharomyces* yeasts to exploit their positive abilities combined with *S. cerevisiae* to avoid stuck fermentations represents a feasible alternative to both spontaneous and inoculated fermentations (**Figure 1**). These combinations could be used to produce wines with unique aromatic characteristics. Based on their capability to produce flavor enhancing enzymes or to modify the concentration of secondary metabolites, different mixed starters have been designed and proposed as a tool to enhance wine quality (**Table 3**). Some of them were designed with the aim of modifying a specific target such as the terpenic profile or final ester concentrations while others show a general impact on wine aroma complexity. The increasing interest in the use of non-*Saccharomyces* yeasts in winemaking has even prompted commercial production of several species including *L. thermotolerans, M. pulcherrima, T. delbrueckii, P. kluyvery*, and *S. pombe*.

**Table 3:** Mixed starters designed to improve primary and secondary wine aroma.

Mixed starter	Impact on wine aroma	Inoculation	Must	Reference
*C. zemplinina/S. cerevisiae*	3MH increase	Co-inoculation	Sauvignon Blanc	Anfang et al., 2009
	Acetic acid decrease	Co-inoculation, sequential	Erbaluce dried grape must, Pinot Grigio	Ciani and Ferraro, 1998; Rantsiou et al., 2012
*D. pseudopolymorphus/S. cerevisiae*	Geraniol, nerol and citronellol increase	Co-inoculation	Chardonnay	Cordero-Otero et al., 2003
*D. vanriji/S. cerevisiae*	Geraniol increase	Sequential	Muscat of Frontignan	García et al., 2002
*H. guilliermondii/S. cerevisiae*	Acetate ester increase	Co-inoculation	Bobal, natural must	Rojas et al., 2003; Moreira et al., 2008
	Sulfur compound increase	Co-inoculation	Natural must	Moreira et al., 2008
*H. uvarum/S. cerevisiae*	Acetate ester increase	Co-inoculation	Synthetic must, Macabeo, natural must	Moreira et al., 2008; Andorrà et al., 2010, 2012
*H. vineae*/ *S. cerevisiae*	Acetate and ethyl ester increase	Co-inoculation, sequential	Bobal, Chardonnay white, Tempranillo	Viana et al., 2009, 2011; Medina et al., 2013
*I. orientalis/S. cerevisiae*	Wine deacidification	Co-inoculation	Campbell’s Early	Kim et al., 2008
*K. gamospora/S. cerevisiae*	Acetate and ethyl ester increase	Sequential	Ribolla	Dashko et al., 2015
*L. thermotolerans/S. cerevisiae*	Wine acidification	Co-inoculation, sequential	Pasteurized natural must, sterile grape must	Kapsopoulou et al., 2007; Comitini et al., 2011; Gobbi et al., 2013
*M. pulcherrima/S. cerevisiae*	α-Terpineol increase	Sequential	Muscat d’Alexandrie	Rodríguez et al., 2010a
	Acetic acid decrease	Co-inoculation	Pasteurized natural must	Comitini et al., 2011
	Ethyl ester increase	Co-inoculation, sequential	Emir, Muscat d’Alexandrie	Zohre and Erten, 2002; Rodríguez et al., 2010a
	Higher alcohol increase	Co-inoculation	Pasteurized natural must	Comitini et al., 2011
*P. fermentans/S. cerevisiae*	Acetic acid decrease	Sequential	Sterile must	Clemente-Jiménez et al., 2005
	Higher alcohol increase	Co-inoculation	Pasteurized natural must	Comitini et al., 2011
*P. kluyveri/S. cerevisiae*	3MHA increase	Co-inoculation	Sauvignon Blanc	Anfang et al., 2009
*S. pombe/S. cerevisiae*	Wine deacidification	Co-inoculation, sequential	Airen, Garnacha	Benito et al., 2013, 2014b
*T. delbrueckii/S. cerevisiae*	α-Terpineol and linalool increase	Sequential	Gewürztraminer	Ĉuŝ and Jenko, 2013
	Acetic acid decrease	Co-inoculation	Botritized Semillon, pasteurized natural must	Bely et al., 2008; Comitini et al., 2011
	Acetate and ethyl ester increase	Co-inoculation, sequential	Sauvignon Blanc, Syrah, Tempranillo	Loira et al., 2014, 2015; Renault et al., 2015
	Higher alcohol increase	Co-inoculation, sequential	Chardonnay, Corvina, Corvinone, Rondinella, pasteurized natural must, Soave, Vino Santo	Comitini et al., 2011; Azzolini et al., 2012, 2015
*W. anomalus/S. cerevisiae*	Acetate and ethyl ester increase	Sequential	Mazuela	Izquierdo-Cañas et al., 2014
*W. saturnus/S. cerevisiae*	Acetate ester increase	Co-inoculation	Emir	Erten and Tanguler, 2010, Tanguler, 2012, 2013
*Z. bailii/S. cerevisiae*	Ethyl ester increase	Co-inoculation	Chardonnay	Garavaglia et al., 2015

When using non-*Saccharomyces* yeasts in mixed starters, there are two general practices of inoculation. The first, known as co-inoculation, involves the inoculation of the selected non-*Saccharomyces* yeasts at high cell concentration together with *S. cerevisiae*, while the second, sequential inoculation, implies that the selected non-*Saccharomyces* yeasts are first inoculated at high levels and allowed to ferment on their own for a given amount of time before *S. cerevisiae* is added to take over the fermentation. Both are feasible practices although potential interactions between yeasts could determine which inoculation strategy is more appropriate.

### Primary Aroma

#### Influence on Terpenes

With the aim of obtaining terpene-enriched wines, *T. delbrueckii, M. pulcherrima, D. hansenii*, and *D. pseudopolymorphus* strains able to produce β-D-glucosidase activity were combined with *S. cerevisiae*.

Higher concentrations of α-terpineol and linalool were found in Gewürztraminer wine fermented with the combination *T. delbrueckii*/*S. cerevisiae*, although more nerol and geraniol were detected in the control fermentation conducted with *S. cerevisiae* alone (Ĉuŝ and Jenko, 2013). Moreover those chemical changes enhanced the overall quality of Gewürztraminer wine.

*Metschnikowia pulcherrima* is known to produce β-D-glucosidase activity able to increase the α-terpineol, nerol as well as geraniol concentrations in monoculture wines (Rodríguez et al., 2010a). However, in wines obtained by mixed fermentation, in either simultaneous or sequential inoculation, nerol and geraniol concentrations were significantly lower than those observed in grape must, and only α-terpineol concentration was higher. This fact was related to the *S. cerevisiae* capability to transform nerol and geraniol into α-terpineol at must pH (Di Stefano et al., 1992; Mateo and Jiménez, 2000), pointing out the relevance of yeast interactions.

With respect to *Debaryomyces* species, a *D. vanriji* strain isolated from grape berry flora was found to influence wine volatiles of the cv. Muscat of Frontignan when co-cultured with native or selected strains of *S. cerevisiae*. The concentrations of several volatiles including terpenols were significantly different between the control and the wines inoculated with *D. vanriji*. The increase in geraniol concentration was attributed to the hydrolysis of the corresponding glucosidic precursor by *D. vanriji* β-D-glucosidase since musts inoculated with the non-*Saccharomyces* yeast showed higher levels of enzymatic activity throughout the fermentation compared to the control sample. Moreover bound geraniol concentration was found to be lower in *D. vanriji* inoculated wines compared to the control one (García et al., 2002). The high terpene concentrations of wines obtained with mixed cultures *D. vanriji*/*S. cerevisiae* was recently confirmed and associated with the production of pectinase, amylase, and xylanase activities along the fermentation (Maturano et al., 2015). Also, a β-D-glucosidase producing *D. pseudopolymorphus* strain when co-cultured with *S. cerevisiae* VIN13 significantly increased concentrations of citronellol, nerol, and geraniol during the fermentation of Chardonnay juice (Cordero-Otero et al., 2003).

#### Influence on Thiols

To take advantage of the capability of *C. zemplinina* to produce volatile thiols, mixed starters of commercially available *S. cerevisiae* strains with *C. zemplinina* isolates were employed in Sauvignon Blanc fermentations. Inoculation with equal amounts or with a ratio that initially favored the non-*Saccharomyces* isolates, produced wines with the greatest increase of the volatile thiol 3MH when compared with the *S. cerevisiae* single ferment. However, the co-ferments with the *C. zemplinina* isolates had significantly lower concentrations of 3MHA (Anfang et al., 2009). By contrast, an elevation in thiol production, especially 3MHA, was found in Sauvignon Blanc wines co-fermented with *P. kluyvery* isolates and different *S. cerevisiae* strains (Anfang et al., 2009). Although both species were able to produce 3MHA in monoculture, the increase in 3MHA concentration observed in co-fermented wines could not be explained under simple additive assumptions suggesting an interaction between the co-fermenting partners. Moreover the elevation in thiols was only seen in co-ferments with certain *S. cerevisiae* strains suggesting that the nature of this particular interaction may not be generalized to the species level. However, the mechanism behind this interaction is still unknown.

Nowadays, selected non-*Saccharomyces* strains for the improvement of wine primary aroma are on the market. A *M. pulcherrima* strain selected for its specific property to release enzymes with α-L-arabinofuranosidase activity is now available. *M. pulcherrima*, with a suitably paired *S. cerevisiae* strain sequentially inoculated, impacts on the expression of terpenes and thiols and it is recommended for Riesling and Sauvignon Blanc wines. Also a commercial product based on a selected strain of *P. kluyveri* is recommended due to its ability to boost fruit flavors through a more efficient conversion of flavor precursors into volatile thiols.

### Secondary Aroma

#### Control of Wine Acidity

Different strategies based on non-*Saccharomyces* yeasts have been described for reducing volatile acidity or either for acidifying or deacidifying wines.

To solve the problem of excessive volatile acidity due to high acetic acid concentrations, non-*Saccharomyces* yeasts such as *T. delbrueckii, M. pulcherrima*, and *C. stellata*/*C. zemplinina* can be employed. *T. delbrueckii*, often described as a low acetic acid producer under standard conditions, retains this quality even fermenting high-sugar media. A mixed culture of *T. delbrueckii* and *S. cerevisiae* was shown to be the best combination for improving the analytical profile of wines produced from botrytized musts, particularly volatile acidity, and acetaldehyde production. Specifically, the mixed *T. delbrueckii*/*S. cerevisiae* culture produced 53% less in volatile acidity and 60% less acetaldehyde than a pure culture of *S. cerevisiae* (Bely et al., 2008). Interestingly, the mixed culture was only effective in simultaneous inoculation since the sequential one resulted in stuck fermentation. Significant reductions in volatile acidity in mixed fermentations of *T. delbrueckii*/*S. cerevisiae* were also reported by other authors (Comitini et al., 2011), who observed the same effect when using *M. pulcherrima*/*S. cerevisiae* starters independently of the inoculum ratios.

The high fermentative capacity of *C. stellata*/*C. zemplinina* has been explored in mixed starters. In a fermentation conducted by a mixture of *C. stellata* and *S. cerevisiae* in Pinot Grigio must (270 g sugars/L), yeast cells were able to completely consume all glucose and fructose while reducing the levels of acetic acid (Ciani and Ferraro, 1998). Similarly, specific strains of *C. zemplinina* when co-inoculated with *S. cerevisiae* were able to reduce the content of acetic acid while maintaining high glycerol and ethanol levels (Rantsiou et al., 2012).

In grape must, different combinations of *Pichia fermentans* with *S. cerevisiae* produced less acetic acid than *S. cerevisiae* in single cultures. Moreover the decrease in acetic acid was accompanied by a substantial increase in aromatic compounds such as acetaldehyde, ethyl acetate, 1-propanol, n-butanol, 1-hexanol, ethyl caprilate, 2,3-butanediol, and glycerol (Clemente-Jiménez et al., 2005).

The ability of non-*Saccharomyces* yeasts to act as acidifying agents is of increasing interest, as global climate change and variations in viticulture and oenology practices have resulted in a trend toward the reduction of the total acidity of wines. *L. thermotolerans*, through the production of L-lactic acid, is a potential acidifying microorganism during must fermentation which could compensate the insufficient acidity of specific grape varieties (Mora et al., 1990; Kapsopoulou et al., 2007). *L. thermotolerans*, in both simultaneous and sequential inoculations with *S. cerevisiae*, provided an effective acidification during alcoholic fermentation although the production of L-lactic acid was dependent on the time of inoculation of the *S. cerevisiae* strain (Kapsopoulou et al., 2007). Also the *L. thermotolerans*/*S. cerevisiae* consortium provoked a pH reduction associated with a significant enhancement in the total acidity and reduction in the volatile acidity, as compared to pure *S. cerevisiae* cultures (Comitini et al., 2011). The mixed fermentations were characterized as well by increases in glycerol and main esters. In agreement with previous results, pH reductions and enhancement of glycerol and 2-phenylethyl alcohol contents were shown in wines fermented with the co-culture. Moreover sensory analysis tests showed significant increases in the spicy notes and in terms of total acidity increases (Gobbi et al., 2013).

Also the deacidifying capacity of *S. pombe* and *Issatchenkia orientalis* (alternative name *Pichia kudriavzevii*) due to the consumption of malic acid has been explored in mixed starters. The combination *S. pombe*/*S. cerevisiae* has proved successful in biological deacidification of white and red wines (Benito et al., 2013, 2014b). In all wines obtained with *S. pombe* either alone or together with *S. cerevisiae*, nearly all the malic acid was consumed, and moderate acetic acid concentrations were formed. Moreover the urea content of these wines was notably lower when compared with those that were made with *S. cerevisiae* alone. White wines obtained with mixed cultures received the best overall scores after sensory evaluation (Benito et al., 2013) but in red wine fermentations, the maximum aroma intensity and quality corresponded to those obtained with *S. pombe* in monoculture (Benito et al., 2014b). Similarly, wines co-fermented by *I. orientalis* and *S. cerevisiae* showed decreased malic acid concentrations and the highest score in sensory evaluation. The co-fermentation also decreased the contents of acetaldehyde, 1-propanol, 2-butanol, and isoamyl alcohol but increased the methanol content (Kim et al., 2008). Recently the capability of *P. kudriavzevii* to degrade malic acid in microvinifications, increasing the pH 0.2–0.3 units, was confirmed (del Mónaco et al., 2014).

Additionally, the combined use of selected *S. pombe* and *L. thermotolerans* strains has been described as a feasible alternative to the traditional malolactic fermentation (Benito et al., 2015). With this approach, malic acid is totally consumed by *S. pombe*, while lactic acid produced by *L. thermotolerans* maintains or increases the acidity of wines produced from low acidity musts. Final wines had more fruity character and they contained less acetic acid and biogenic amines than the traditional malolactic fermentation controls (Benito et al., 2015).

#### Influence on Esters

The increase of fruity acetate esters has been the main target of mixed starters designed with *Hanseniaspora* species. *H. guilliermondii* and *H. uvarum* grown as mixed cultures with *S. cerevisiae* in grape must increased the 2-phenylethyl acetate and isoamyl acetate content of wines, respectively (Rojas et al., 2003; Moreira et al., 2008). However, the excessive production of ethyl acetate limited the applicability of both mixed starters. Similarly, although a desirable increase of acetate esters in mixed fermentations of Macabeo and synthetic must with *H. uvarum*/*S. cerevisiae* was reported, the high concentration of acetic acid hampered the industrial application of the mixed starter (Andorrà et al., 2010, 2012). However, since a large strain variability is associated to metabolite production (Plata et al., 2003; Romano et al., 2003; Ciani et al., 2006), the excessive concentration of both ethyl acetate and acetic acid can be avoided by means of specific screenings. In this regard a *H. vineae* strain yielding high levels of 2-phenylethyl acetate while producing levels of acetic acid and ethyl acetate within the optimal ranges described for wine was selected (Viana et al., 2008). Moreover the potential of using that selected strain in a mixed starter with *S. cerevisiae* to increase the levels of 2-phenylethyl acetate in wines without compromising quality was demonstrated (Viana et al., 2009). Besides, the ratio of both yeast strains in the mixed culture modulated ester concentrations leading to wines with a wide range of flavor compounds. Further studies showed that the selected *H. vineae* strain inoculated as a part of a sequential mixed starter was able to compete with native yeasts present in a non-sterile must and modify the wine aroma profile, specifically 2-phenylethyl acetate concentration (Viana et al., 2011). Recently, wines obtained from industrial Chardonnay white grape vinifications conducted by sequential *H. vineae*/*S. cerevisiae* inoculation showed a significant increase in fruity intensity described as banana, pear, apple, citric fruits, and guava, in comparison to spontaneous and pure *S. cerevisiae* fermentations. Fruity intensity was mainly correlated to higher concentrations of acetyl and ethyl esters and relative decreases in alcohols and fatty acids (Medina et al., 2013).

An increase in isoamyl acetate content can also be achieved by fermenting Emir must with *Williopsis saturnus*/*S. cerevisiae* cultures. Furthermore the mixed culture did not produce any off-flavors, although the changes observed in the aromatic profile of mixed wines were inoculum and temperature dependent (Erten and Tanguler, 2010; Tanguler, 2012, 2013). Wines elaborated by sequential fermentation of *W. anomalus* and *S. cerevisiae* presented higher levels of acetate and ethyl esters and of linear alcohols, which contribute to increase the aromatic quality with floral and fruity notes (Izquierdo-Cañas et al., 2014). With the aim of obtaining white wines with an enhanced aromatic complexity, a *Zygosaccharomyces bailii* strain characterized as producer of several esters (Garavaglia et al., 2014) was inoculated together with *S. cerevisiae*. In all trials that contained the non-*Saccharomyces* yeast the production of ethyl esters was increased in comparison to the vinification control (Garavaglia et al., 2015).

In mixed cultures *T. delbrueckii*/*S. cerevisiae* the formation of specific esters was reported. For instance, the mixed culture produced Tempranillo wines with larger quantities of 2-phenylethyl acetate and ethyl lactate than single *S. cerevisiae* fermentations (Loira et al., 2014), or larger amounts of isoamyl acetate, hexyl acetate, ethyl hexanoate, and ethyl octanoate in Syrah wines (Loira et al., 2015). Also, ethyl propanoate, ethyl isobutanoate, ethyl dihydrocinnamate, isobutyl acetate, and isoamyl acetate concentrations were increased in wines obtained by mixed fermentations *T. delbrueckii*/*S. cerevisiae*, either in sequential or simultaneous inoculation (Renault et al., 2015). Favoring *T. delbrueckii* development when performing sequential inoculation enhanced the concentration of the above mentioned ethyl esters, which were linked to *T. delbrueckii* activity. On the contrary, simultaneous inoculation restricted the growth of *T. delbrueckii*, limiting the production of its activity markers. However, simultaneous inoculation involved a high production of numerous esters due to more important positive interactions between yeast species. These results suggested that the ester concentration enhancement via interactions during mixed modalities was due to *S. cerevisiae* production in response to the presence of *T. delbrueckii* (Renault et al., 2015). A pure culture of *T. delbrueckii* selected for its properties to enhance wine aromatic and mouthfeel complexity is available on the market. When used in sequential inoculation with compatible selected *S. cerevisiae*, it favors the perception of certain esters without overwhelming the wines.

Finally, aromatic complexity of Ribolla wines can be improved by sequential fermentation with *K. gamospora* and *S. cerevisiae* due to the enhanced production of esters such as 2-phenylethyl acetate and ethyl propionate and also of 2-phenylethyl alcohol (Dashko et al., 2015).

#### Influence on Higher Alcohols

The presence of *T. delbrueckii* in mixed starters has been associated with increases in the production of 2-phenylethyl alcohol in different kinds of wine. Mixed starters *T. delbrueckii*/*S. cerevisiae* were proposed for the production of Amarone wine, a high-alcohol dry red wine obtained from withered grapes. The most significant changes caused by the presence of *T. delbrueckii* were observed among alcohols, specifically benzyl alcohol and 2-phenylethyl alcohol, but also in fermentative esters, fatty acids and lactones, which are important in the Amarone wine flavor (Azzolini et al., 2012). Interestingly the increase in the levels of 2-phenylethyl alcohol seemed to be related to the β-glucosidase activity of the *T. delbrueckii* strain employed, although other factors could not be discarded. Also in the fermentation of dry and sweet wines, mixed cultures *T. delbrueckii*/*S. cerevisiae* affected the content of several important volatile compounds, including 2-phenylethyl alcohol, isoamyl acetate, fatty acid esters, C_4_–C_10_ fatty acids and vinylphenols (Azzolini et al., 2015). In addition to *T. delbrueckii* mixed starters, *M. pulcherrima*/*S. cerevisiae, L. thermotolerans*/*S. cerevisiae*, and *K. gamospora*/*S. cerevisiae* fermentations resulted in higher productions of 2-phenylethyl alcohol (Comitini et al., 2011; Dashko et al., 2015).

#### Triple Mixed Cultures

Finally, and with the aim of mimicking the complex yeast microbiota present in fermenting musts, wine mixed cultures composed by more than one non-*Saccharomyces* species in combination with *S. cerevisiae* have been also developed. However, the number of studies focused on wine aroma development is still low and results somehow controversial.

In this regard, fermentations of natural grape musts with a *Saccharomyces* strain together with a *C. zemplinina* and/or a *H. uvarum* strains showed the preferential use of some groups of amino acids (aliphatic, aromatic, and sulphur amino acids) in the mixed fermentations compared with the pure cultures. These results suggested that the presence of several yeast species might improve the uptake or consumption of some amino acids by some kind of synergistic mechanism (Andorrà et al., 2010). However the preferential use of amino acids did not have a clear consequence on aroma production as it would be expected: fermentations with the triple mixed culture only stood out for ethyl lactate production and significant differences were only observed with the pure *S. cerevisiae* fermentation, while fermentations including one or two non-*Saccharomyces* strains were comparable to each other. Moreover the amount of acetic acid was well above the admissible levels and thus compromising the immediate application of these mixed cultures (Andorrà et al., 2010). Later on, the same authors reported significant differences in the above results when a synthetic grape must was used (Andorrà et al., 2012).

Production of white wine by sequential inoculation of *H. anomala, T. delbrueckii*, and *S. cerevisiae* has been proposed (Izquierdo-Cañas et al., 2011) Resulting wines were chemically different to those produced by *S. cerevisiae* alone, by *H. anomala*/*S. cerevisiae*, or by *T. delbrueckii*/*S. cerevisiae*. Surprisingly, the two last combinations produced wines exhibiting more complexity than the one including the two non-*Saccharomyces* species, which in fact was comparable to the pure *S. cerevisiae* fermentation.

Nowadays, a blend of three yeasts, *S. cerevisiae, K. thermotolerans*, and *T. delbrueckii*, is commercialized. The mixture gives tropical fruitiness and an overall aromatic intensity in white wines and more pronounced fruity and spicy notes in red wines.

## Final Considerations

Based on numerous studies showing the positive influence of non-*Saccharomyces* yeasts in winemaking, the wine industry has been directed toward the use of controlled mixed fermentations. Indeed designed mixed starters with selected non-*Saccharomyces* strains and *S. cerevisiae* can enhance, as pointed out in this review, primary and secondary wine aroma, but also they are involved in reductions of the ethanol content of wine (González et al., 2013; Contreras et al., 2015; Morales et al., 2015), control of the spoilage wine microflora (Oro et al., 2014), release of mannoproteins (Domizio et al., 2014) or wine color stabilization (Morata et al., 2012; Loira et al., 2015). Moreover they can exert a positive effect in base wines for sparkling wine production improving foaming properties (González-Royo et al., 2015). Remarkably, a new red winemaking technology based on the combined use of two non-*Saccharomyces* yeast strains has been developed as an alternative to the traditional malolactic fermentation (Benito et al., 2015).

In addition to the mandatory strain selection, the benefits of mixed cultures should be tested in different grape musts since different nutritional characteristics and limitations might modify the impact of the individual components of the starter on the final wine. Moreover mixed cultures should be tested at industrial or semi-industrial scales because it has been reported that the production of different metabolites can vary depending on the fermentation volume and the oxygen conditions (Beltran et al., 2008; Viana et al., 2009). Certainly the study of the impact of common oenological practices on the dynamics of non-*Saccharomyces* yeasts will be also useful for a better management of mixed fermentations (Albertin et al., 2014).

Considering that the main reason for re-evaluating non-*Saccharomyces* yeasts and for introducing mixed cultures in the winemaking process was to get differentiated wines reflecting the characteristic of a given wine region, the commercial assortment of non-*Saccharomyces* cultures is still reduced. In this context the continuous ecological studies as well as the oenological and sensory characterization of autochthonous non-*Saccharomyces* and even *S. cerevisiae* isolates will provide appropriate candidates to be included as a part of commercial mixed starter cultures for the production of typical wines (Canonico et al., 2015; Teixeira et al., 2015).

Finally, rational design of mixed cultures should take into account not just results from smart screenings that allow exploiting positive features of non-*Saccharomyces* yeasts but also potential interactions among microorganisms. Some yeast interactions reported to occur in mixed starters have been briefly discussed in this review, but little is known about the mechanisms involved. In fact, positive, negative, and neutral interactions in mixed fermentations of non-*Saccharomyces* and *Saccharomyces* yeasts for the formation of aromatic compounds have been identified (Sadoudi et al., 2012). These interactions seem to be strain-dependent for both non-*Saccharomyces* and *S. cerevisiae* strains (Anfang et al., 2009; Canonico et al., 2015) and might affect the entire metabolic pathway. Current knowledge on wine yeast interactions has been recently revised (Ciani and Comitini, 2015) but it is an area that requires in-depth studies. Undoubtedly the application of high throughput techniques will offer a powerful approach for unraveling microbial interactions and thus it will allow a better design of mixed cultures and also an increased control over mixed culture fermentations.

## Author Contributions

BP, JG, and PM made contributions to conception and design of the review. BP and JG draft the manuscript. PM supervised and edited the manuscript. All authors commented on the manuscript at all stages.

## Conflict of Interest Statement

The authors declare that the research was conducted in the absence of any commercial or financial relationships that could be construed as a potential conflict of interest.
